# Long-lasting alterations in adipose tissue density and adiponectin production in people living with HIV after thymidine analogues exposure

**DOI:** 10.1186/s12879-019-4347-y

**Published:** 2019-08-09

**Authors:** Marco Gelpi, Andreas Dehlbæk Knudsen, Karoline Brostrup Larsen, Amanda Mocroft, Anne-Mette Lebech, Birgitte Lindegaard, Jens Lundgren, Klaus Fuglsang Kofoed, Susanne Dam Nielsen, on behalf of the Copenhagen Comorbidity in HIV Infection (COCOMO) Study

**Affiliations:** 1 Viro-immunology Research Unit, Department of Infectious Diseases 8632, Rigshospitalet - University of Copenhagen, Blegdamsvej 9B, DK-2100 Copenhagen Ø, Denmark; 2 HIV Epidemiology and Biostatistics Unit, Department of Infection and Population Health, UCL, London, UK; 3 Center for inflammation and Metabolism, Rigshospitalet, Copenhagen, Denmark; 4 Department of pulmonary and infectious diseases, Nordsjællands Hospital, Hillerød, Denmark; 5 CHIP, Department of Infectious Diseases 8632, Rigshospitalet, University of Copenhagen, Copenhagen, Denmark; 6 Department of Cardiology, Rigshospitalet, University of Copenhagen, Copenhagen, Denmark; 7 Department of Radiology, Rigshospitalet, University of Copenhagen, Copenhagen, Denmark

**Keywords:** HIV, Visceral fat, Adipose tissue function, Adiponectin, Adipocytes

## Abstract

**Background:**

Thymidine analogues (TA) and didanosine (ddI) are associated with long-lasting adipose tissue redistribution. Adiponectin is a widely used marker of adipocyte activity, and adipose tissue density assessed by CT-scan is associated with adipocyte size and function. We hypothesized that prior exposure to TA and ddI was associated with long-lasting adipose tissue dysfunction in people living with HIV (PLWH). Thus, we tested possible associations between markers of adipose tissue dysfunction (adipose tissue density and adiponectin) and prior exposure to TA and/or ddI, years after treatment discontinuation.

**Methods:**

Eight hundred forty-eight PLWH from the COCOMO study were included and stratified according to prior exposure to TA and/or ddI (with, *n* = 451; without *n* = 397). Visceral (VAT) and subcutaneous (SAT) adipose tissue area and density were determined by single slice abdominal CT-scan at lumbar 4th level. Venous blood was collected and analyzed for adiponectin. Multivariable linear and logistic regression analyses were used to test our hypotheses. Multivariable models were adjusted for age, sex, smoking, origin, physical activity, BMI, and adipose tissue area (VAT or SAT area, accordingly to the outcome).

**Results:**

prior exposure to TA and/or ddI was associated with excess risk of low VAT (adjusted OR (aOR) 1.74 [1.14; 2.67]) and SAT density (aOR 1.74 [1.18; 2.58]), for a given VAT and SAT area, respectively. No association between VAT and SAT density with time since TA and/or ddI discontinuation was found. 10 HU increase in VAT density was associated with higher adiponectin plasma level and this association was not modified by prior exposure to TA and/or ddI. Prior exposure to TA and/or ddI was associated with 9% lower [− 17;-2] plasma adiponectin levels and with excess risk of low adiponectin (aOR 1.74 [1.10; 2.76]).

**Conclusions:**

We described low adipose tissue density and impaired adiponectin production to be associated with prior exposure to TA and/or ddI even years after treatment discontinuation and independently of adipose tissue area. These findings suggest that prior TA and ddI exposure may have long-lasting detrimental effects on adipose tissue function and, consequently, on cardiometabolic health in PLWH.

**Electronic supplementary material:**

The online version of this article (10.1186/s12879-019-4347-y) contains supplementary material, which is available to authorized users.

## Introduction

Thymidine analogues (TA) and didanosine (ddI) have known deleterious effect on adipose tissue metabolism [1]. Previous results from our group suggested that prior use of TA and ddI in people living with HIV (PLWH) is associated with alterations in body fat distribution, particularly loss of subcutaneous (SAT) and accumulation of visceral adipose tissue (VAT) [1], even years after treatment discontinuation. This phenotype is known to be associated with increased of cardiometabolic risk.

Unfavorable changes in adipose tissue function and quality accompanies ectopic fat deposition and are strongly associated with increased incidence of cardiovascular diseases in uninfected individuals [2]. Recently, the association between computed tomography-measured adipose tissue density (measured in Hounsfield Units (HU)) and both adipocytes size and function has been described [3–6]. Low adipose tissue density has been suggested to indicate adipocyte hypertrophy and is associated with reduced secretory capabilities, both of which are considered hallmarks of adipose tissue dysfunction and increased cardiovascular risk [3, 4, 7, 8].

While it was previously only considered an energy storage, adipose tissue has drawn increasing attention as endocrine organ and as a fundamental crossway in inflammation control [9]. Alterations in plasma levels of adipokines follows adipose tissue dysfunction, and are associated with increased cardiovascular risk [3].

Our aim was to investigate if alterations in fat distributions found in PLWH with prior exposure to TA and/or ddI are accompanied by long-lasting markers of adipose tissue dysfunction even years after treatment discontinuation. Previous studies investigating adipose tissue quality in PLWH have relied on biopsy and invasive procedures and included a small number of participants. In the present study, we tested the hypotheses that: i) for a given VAT and SAT area, prior use of TA and/or ddI is associated with low VAT and SAT density, respectively; ii) VAT area and density are associated with adiponectin plasma levels and these associations are modified by prior exposure to TA and/or ddI; iii) for a given VAT area, prior use of TA and/or ddI is associated with excess risk of low adiponectin.

## Methods

The Copenhagen comorbidity in HIV infection (COCOMO) study is a longitudinal study with the aim of assessing the burden of non-AIDS comorbities in PLWH. Of the 1099 PLWH enrolled in the COCOMO study, 848 PLWH with a CT-scan were included. Procedures for recruitment and data collection have been described in detail elsewhere [10].

Ethical approval was obtained by the Ethics Committee (H-15017350). Written informed consent was obtained from all participants.

### Clinical assessments

Structured questionnaires were used to collect information about demographics, physical activity, and smoking. Data regarding HIV infection were obtained from review of medical charts [10].

All physical examinations were performed by clinic staff as previously described [10].

### Outcome definitions

Low adiponectin was defined as having adiponectin plasma levels in the lowest quartile. Similarly, low VAT and SAT density were defined as having VAT and SAT density, respectively, in the lowest quartile.

### Blood samples

Non-fasting venous blood was collected and analyzed for adiponectin at Herlev Hospital, Copenhagen [10].

### CT-scan measurement

CT imaging was performed using 320-multidetector scanner (Aquilion One ViSION Edition, Canon, Japan) in a single rotation (275 ms). Field of view (FOV) was 500, tube voltage was 120 kVp and current was 210 mA (independent of BMI). For measurement of visceral and subcutaneous adipose tissue, an 8 mm section (2 × 4.0 mm) was reconstructed centered at the level of the 4th lumbar vertebra. Trained personnel used commercially available CT software (Fat Measurement, Aquilion ONE; Canon, Japan) to measure the cross-sectional area of adipose tissue defined as voxels with attenuation values in the range of − 150 to − 70 Hounsfield units. From within a manually adjusted region of interest delineated by the muscular compartments, VAT area was automatically calculated. SAT was defined as adipose tissue superficial to the abdominal and paraspinal muscles. Intraintestinal and intramuscular adipose tissues were manually excluded. Mean density for VAT and SAT, respectively, were calculated and reported, using four regions of interest within each fat depot. All measurements were performed by a single investigator blinded to stratification status. Intra-reader variability evaluation showed high intra-reader correlation (0.975 for VAT density, 0.973 for SAT density) and low mean intra-reader absolute difference (1.01, SD 0.77 for VAT density; 1.02, SD 0.99 for SAT density).

### Statistical analysis

Continuous variables were reported as median and interquantile range [IQR] and categorical variables as frequency and percentage (%). Different groups were compared with t-tests or Mann Whitney U test for continuous data with normal or non-normal distribution, respectively, and chi square/Fisher’s tests for categorical data. In regression analyses, adiponectin levels were log-transformed.

Associations between prior exposure to TA and/or ddI and our outcomes were explored with linear or logistic regression models for continuous or binary outcomes, respectively. Β coefficients or odds ratio, respectively, and 95% confidence intervals [CIs] were computed and reported after adjustment for a base model, which included age, sex, smoking, origin, physical activity, BMI, and fat area (VAT or SAT area, accordingly to the outcome) as covariates.

Associations between our outcomes and the cumulative period of exposure to and the cumulative time since discontinuation of TA and/or ddI were explored by adding these covariates into the base model. These analyses included only individuals with prior exposure to TA and/or ddI.

Possible interactions of exposure to TA and/or ddI with VAT area and density, respectively, were tested in multivariable linear analysis models with plasma adiponectin levels as dependent variable.

Sensitivity analyses considering only individuals > 40 years of age were performed.

Due to log-transformation of plasma adiponectin levels, regression results regarding this outcome are reported as changes in percentage.

*P*-values < 0.05 were considered statistically significant. Statistical analyses were performed using R 3.4.1 (R Foundation, Austria).

## Results

### Demographics and HIV-specific characteristics

Eight hundred forty-eight PLWH from the COCOMO study were included and 451 (53.1%) had current or prior exposure to TA and/or ddI. Of those, 445 (98.6%) had only prior exposure, and 6 (1.4%) were still exposed. The mean cumulative exposure period to TA and/or ddI was 6.6 (SD, 4.2) years and mean time since discontinuation was 9.4 (SD, 2.7) years. Demographics and HIV-specific characteristics are depicted in Table 1.

**Table 1:** Study groups characteristics

	Without exposure to TA and/or ddI (*n* = 397)	With exposure to TA and/or ddI (*n* = 451)	*p*-value
Age, mean (sd)	46.1 (10.5)	55.2 (10.2)	< 0.0001
Sex, male, *n* (%)	361 (90.9)	373 (82.7)	0.0006
Origin, n (%)			0.0497
Scandinavian	274 (70.3)	343 (76.9)	
Other EU	49 (12.6)	44 (9.9)	
Middle East and Indian subcontinent	13 (3.3)	5 (1.1)	
Other	54 (13.8)	54 (12.1)	
BMI Categories, *n* (%)			0.4838
<18.5	9 (2.3)	11 (2.5)	
18.5–24.9	211 (53.3)	232 (51.8)	
25–29.9	145 (36.6)	156 (34.8)	
30	31 (7.8)	49 (10.9)	
Smoking status, *n* (%)			< 0.0001
Never smoker	162 (41.8)	136 (30.8)	
Current smoker	119 (30.7)	112 (25.3)	
Former smoker	107 (27.6)	194 (43.9)	
Physical activity, *n* (%)			0.0709
Very inactive	36 (9.6)	38 (8.8)	
Moderately inactive	117 (31.1)	161 (37.4)	
Moderately active	161 (42.8)	184 (42.8)	
Very active	62 (16.5)	47 (10.9)	
Exposure to thymidine analogues and didanosine, *n* (%)			–
Prior exposure	–	445 (98.6)	
Current exposure	–	6 (1.4)	
Mode of HIV transmission, *n* (%)			0.0032
Heterosexual	64 (16.2)	100 (22.4)	
IDU	5 (1.3)	5 (1.1)	
MSM	311 (78.9)	306 (68.6)	
Other	14 (3.6)	35 (7.8)	
CD4 nadir < 200 cells, *n* (%)	89 (23.2)	258 (57.8)	< 0.0001
Viral load < 50, *n* (%)	369 (93.9)	436 (97.1)	0.0357
Current cART, *n* (%)	397 (100.0)	449 (99.8)	1
VAT area, cm^2^, median [iqr]	62.3 [32.2, 109.2]	106.5 [55.8, 166.5]	< 0.0001
VAT density, HU, median [iqr]	-108.9 [− 113.6, − 104.9]	− 113.9 [− 115.7, − 109.7]	< 0.0001
SAT, cm^2^,median [iqr]	137.9 [88.6, 198.2]	126.2 [81.2, 177.2]	0.0768
SAT density, HU, median [iqr]	− 110.4 [− 112.7, − 106.0]	−111.1 [− 113.7, − 108.1]	0.0002
Adiponectin, μg/ml, median [iqr]	11.8 [8.7, 14.7]	11.5 [8.1, 16.3]	0.4822
Low adiponectin, *n* (%)	74 (23.3)	105 (28.8)	0.1173

Abbreviations: visceral adipose tissue, VAT; subcutaneous adipose tissue, SAT; body mass index, BMI; standard deviations, SD; intravenous drug use, IDU; male-to-male sex, MSM, combined antiretroviral therapy, cART thymidine nucleoside analog reverse-transcriptase inhibitors, TA; Hounsfield units, HU

### Visceral adipose tissue density

**Fig. 1:**
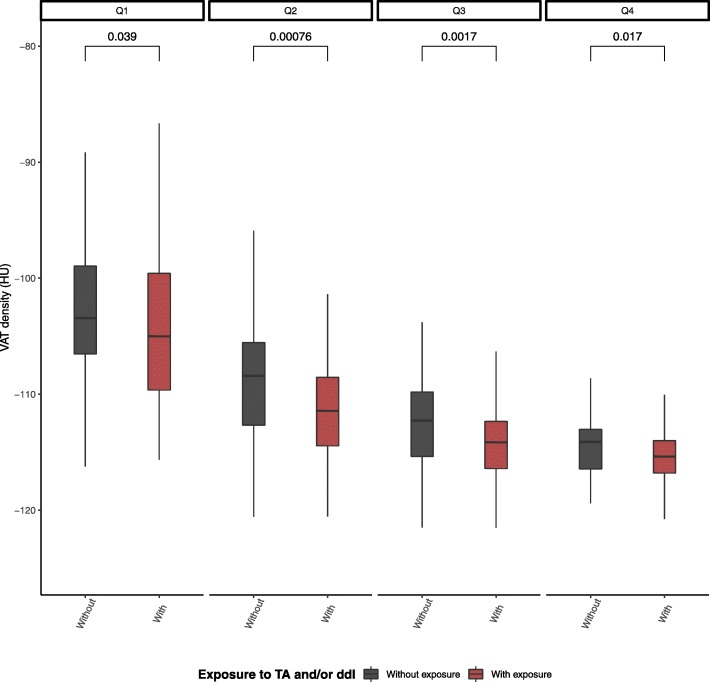
Comparison of visceral adipose tissue density in people living with HIV with and without prior exposure to TA and/or ddI stratified in quartiles of VAT. Differences in VAT density between PLWH with and without exposure to TA and/or ddI after stratification of participants according to quartiles of VAT area. Abbreviations: people living with HIV, PLWH; thymidine nucleoside analog reverse-transcriptase inhibitors, TA; didanosine, ddI; visceral adipose tissue, VAT; Hounsfield unit, HU; quartiles 1–4, Q1–4

PLWH with exposure to TA and/or ddI had lower VAT density than PLWH without (− 113.9 [− 115.7 - -109.7] vs. -108.9 HU [− 113.6 - -104.9], *p*-value < 0.0001). This finding was reproduced also after stratification according to quartiles of VAT (Fig. 1). Prior exposure to TA and/or ddI was associated excess risk of low VAT density, both before (crude OR 2.76 [1.97; 3.87]) and after adjusting for confounders (aOR 1.74 [1.14; 2.67]) (Table 2). This association was found to be consistent in linear models (Additional file 1: Table S1). In PLWH with exposure to TA and/or ddI, no association between VAT density and either cumulative exposure period to or time since discontinuation of these treatments was found (Table 3).

**Table 2:** Association of prior exposure to TA and/or ddI with low VAT and SAT density

	Low visceral adipose tissue density		Low subcutaneous adipose tissue density	
	Crude OR [95% CI]	Adjusted OR [95% CI]*	Crude OR [95% CI]	Adjusted OR [95% CI]**
Prior exposure to TA and/or ddI	2.76 [1.97; 3.87]	1.74 [1.14; 2.67]	1.61 [1.17; 2.22]	1.74 [1.18; 2.58]
Age, per 10 years	1.78 [1.53; 2.07]	1.23 [1.01; 1.50]	1.23 [1.07;1.42]	1.17 [0.98; 1.40]
Sex, male	2.21 [1.25; 3.90]	1.73 [0.84; 3.57]	0.84 [0.53; 1.31]	2.90 [1.57; 5.35]
VAT area, per 50cm^2^ increase	2.18 [1.91; 2.49]	1.89 [1.59; 2.25]	–	–
SAT area, per 50cm^2^ increase	–	–	1.28 [1.16; 1.42]	2.00 [1.64; 2.43]

Abbreviations: visceral adipose tissue, VAT; subcutaneous adipose tissue, SAT; thymidine nucleoside analog reverse-transcriptase inhibitors, TA; didanosine, ddI. Multivariable models were adjusted for: age, sex, origin, physical activity, BMI, smoking, VAT* (or SAT**) area, and prior exposure to TA and/or ddI

**Table 3:** Association between cumulative periods of exposure to and time since discontinuation of TA and ddI with adipose tissue density

	VAT density	*p*-value	SAT density	*p*-value
	Adjusted β* [95% CI]		Adjusted β** [95% CI]	
Cumulative time of exposure to TA and/or ddI				
<3.6 years	Ref		Ref	
3.6–6.3 years	1.0 [−0.5;2.5]	0.198	−1.0 [−2.4;0.3]	0.141
6.4–9.2 years	0.2 [− 1.3;1.8]	0.770	−2.8 [−4.2;-1.4]	< 0.001
>9.2 years	− 0.5 [− 2.1;1.2]	0.578	−2.3 [−3.8;-0.9]	0.001
Time since discontinuation of TA and/or ddI				
<8.1 years	Ref		Ref	
8.1–9.6 years	−0.3 [− 1.8;1.2]	0.702	−0.0 [− 1.4;1.4]	0.965
9.7–10.7 years	1.4 [− 0.1;2.9]	0.074	0.4 [− 1.1;1.8]	0.593
>10.7	0.6 [− 1.0;2.1]	0.460	−0.6 [− 2.0;0.9]	0.431

β coefficients represent the degree of change in HU of VAT and SAT, respectively, associated with each level of the explanatory variables

Abbreviations: visceral adipose tissue, VAT; subcutaneous adipose tissue, SAT; thymidine nucleoside analog reverse-transcriptase inhibitors, TA; confidence interval, CI

All the models were adjusted for age, sex, origin, physical activity, smoking, BMI, cumulative time of exposure to TA and/or ddI, time since discontinuation of TA and/or ddI, VAT area* (or SAT area**)

### Subcutaneous adipose tissue density

PLWH with exposure to TA and/or ddI had slightly lower SAT density (− 111.1 HU [− 113.7 - -108.1] vs. -110.4 HU [− 112.7 - -106.0]; *p*-value 0.0002). This difference was more evident in linear regression models after adjusting for, among the others, SAT area (Additional file 1: Table S1). Accordingly, prior exposure to TA and/ddI was associated with excess risk of low SAT density, both before (crude OR 1.61 [1.17; 2.22]) and after adjusting for confounders (aOR 1.74 [1.18; 2.58]) (Table 2). This association was found to be consistent in linear models (Additional file 1: Table S1). Longer periods of cumulative exposure to TA and/or ddI were associated with lower SAT density (Table 3). No association between time since discontinuation of these treatments and SAT density was found (Table 3).

### Plasma adiponectin levels

No difference in plasma adiponectin levels and in prevalence of low plasma adiponectin was found between PLWH with and without exposure (Table 1). After adjusting for confounders, prior exposure to TA and/or ddI was associated with 9% lower [− 17; − 2] plasma adiponectin levels and with excess risk of low plasma adiponectin (aOR 1.74 [1.10; 2.76]). Borderline interaction between prior exposure to TA and/or ddI and VAT area on adiponectin plasma levels was found (p-interaction 0.063).

For a given VAT area, 10 HU increase in VAT density was associated with 12% [1; 23] and 9% [− 1; 22] higher plasma adiponectin levels in PLWH with and without exposure to TA and/or ddI, respectively, even though not statistically significant in the latter. The association between VAT density and adiponectin levels was not modified by prior exposure to TA and/or ddI (p-interaction 0.778). No association between SAT density and plasma adiponectin levels was found in the two groups.

### Sensitivity analyses

All results were consistent when limiting the analyses to participants > 40 years old.

## Discussion

In the present study, prior exposure to TA and/or ddI was associated with excess risk of low adipose tissue density and low plasma adiponectin irrespective of fat quantity. These findings suggest that long-lasting alterations of fat distribution previously described in PLWH with prior exposure to TA and/or ddI [1] are accompanied by markers of adipose tissue dysfunction, even years after treatment discontinuation.

TA analogues were among the first antiretroviral treatments introduced to face the HIV epidemic. While effective in helping to control viral replication, they were characterized by severe adverse effects on adipose tissue, whose reversibility is still a subject of debate [1, 11]. While the current use of TA and ddI has declined worldwide, TA was one of the recommended initial antiretroviral treatments until 2005 [12] and any lasting detrimental effects will continue to affect a large proportion of PLWH. Accordingly, increased risk of abdominal obesity and visceral adipose tissue accumulation has been recently described in PLWH, especially in those with prior exposure to TA and/or ddI [1, 13].

The accumulation of adipose tissue can be characterized by hyperplasia (increase in number of cells) or hypertrophy (increase in cell size) [7]. While the former is accompanied by well-preserved adipocyte function, adipocyte hypertrophy is associated with increased lipid content and decreased secretory function [7]. Adipocytes size is inversely associated with adipose tissue density, measured by CT-scan [3, 8, 14]. Previous studies described lower adipose tissue density to be associated with adverse cardiovascular risk in uninfected individuals [2, 3, 5, 8]. In the present study, for a given VAT and SAT area, prior use of TA and/or ddI was associated with lower adipose tissue density. This finding may suggest that adipocytes hypertrophy, rather than hyperplasia, characterizes adipose tissue accumulation in individuals who had been exposed to these treatments. The lack of association between adipose tissue density and time since discontinuation of TA and/or ddI may further suggest irreversible effect of these treatments on adipose tissue density, at least in the time-period considered in the present study.

Mitochondria are fundamental in adipocyte maturation [7]. Mild mitochondrial dysfunction leads to a moderate increase in reactive oxygen species (ROS) production and to reduction in mitochondrial DNA, causing reactive hypertrophy in the affected adipocytes [7]. We speculate that TA and ddI-associated mitochondrial toxicity may lead to mild mitochondria dysfunction in VAT adipocytes, and, consequently, to hypertrophy, that is present years after treatment discontinuation. This hypothesis is in line with previous studies describing long-lasting mitochondrial toxicity after TA exposure [15, 16]. Interestingly, in the SAT compartment, we found prior exposure to TA and/or ddI to be associated with lower adipose tissue density in the presence of smaller SAT area compared to those without exposure. We speculated that in SAT, characterized by lower resistance to mitochondrial toxicity, the same events may lead to severe mitochondrial dysfunction, severe hypertrophy and, eventually, to the loss of adipocytes [7], which may at least partly explain the co-presence of low SAT density and smaller SAT area in PLWH with exposure to TA and/or ddI.

Reduction in adipose tissue density and mitochondrial dysfunction are also associated with reduction of adipocytes’ secretory capabilities [6, 17]. Accordingly, in our population we found a direct association between VAT density and plasma adiponectin levels. However, this association was not modified by prior exposure to TA and/or ddI. Low plasma adiponectin is associated with increased systemic inflammation and CVD risk in both uninfected individuals and PLWH [18]. In particular, adiponectin has been previously described to have antioxidant, anti-inflammatory properties and to beneficially affect insulin resistance [19]. In the present study, prior use of TA and/or ddI was associated with excess risk of low plasma adiponectin. While the inverse association between VAT accumulation and adiponectin production is well-known, we present novel data suggesting that prior exposure to TA and/or ddI is associated with significantly reduced adipocyte secretory capabilities even in individuals with comparable visceral fat quantity and years after treatment discontinuation. The interaction between prior exposure to TA and/or ddI with VAT area on adiponectin levels, while only borderline significant, may further suggest a detrimental effect of TA and/or ddI on adipocyte secretory function following adipose tissue accumulation.

Taken together our data suggest the potential irreversibility of harmful effects of TA and/or ddI on adipose tissue function, which may negatively affect CVD risk in PLWH with prior exposure to these treatments. Increasing evidence suggest adipose tissue dysfunction to have a primary role in the development of CVD through both direct and indirect mechanisms [8]. Accordingly, dysfunctional adipocytes have been described to promote systemic inflammation, insulin resistance and atherosclerosis due to, among the others, reduced production of bioactive antioxidant and anti-inflammatory adipokines, such as adiponectin [19], and increased production of pro-thrombotic molecules [20] and pro-inflammatory cytokines [8].

The present study has several limitations. Due to the cross-sectional design of the present study, no conclusion on causality can be drawn. Differences in age, sex, and adipose tissue area between the two groups may explain part of the findings. However, a possible confounding effect of these variables was reduced by adjusting for, among the others, age, sex, and adipose tissue area in multivariable analyses. Finally, the lack of histologic samples prevented from investigating the association between cell morphology and CT-findings in the individuals included in the present study and from evaluating the impact of non-adipocyte cell populations on adipose tissue density.

## Conclusions

In conclusion we described excess risk of low adipose tissue density and adiponectin production in PLWH with prior use of TA and/or ddI. These results may suggest that long-lasting adipose tissue dysfunction accompanies alterations in fat distribution, even years after TA and/or ddI discontinuation. Given the central role of adipose tissue in the regulation of metabolism, cell morphology and function analyses may be warranted in order to confirm possible long-lasting detrimental effects of prior TA and ddI exposure on adipose tissue function and, consequently, on cardiometabolic health in PLWH.

## Additional file

Additional file 1: **Table S1.** Linear Regression Model predicting the degree of change (with 95% CI) in HU of VAT and SAT density. (DOCX 14 kb)

## Data Availability

All raw data are available by request to corresponding author.

## Abbreviations

ddI: didanosine
PLWH: people living with HIV
SAT: subcutaneous adipose tissue
TA: thymidine analogues
VAT: visceral adipose tissue
