# Supplementary material for: Long-lasting alterations in adipose tissue density and adiponectin production in people living with HIV after thymidine analogues exposure
Source: BMC Infect Dis. 2019 Aug 9;19:708. doi: 10.1186/s12879-019-4347-y (PMC6689174; doi:10.1186/s12879-019-4347-y)
Supplement: Supplementary file 1 — Table S1. Linear Regression Model predicting the degree of change (with 95% CI) in HU of VAT and SAT density. (DOCX 14 kb) [file 12879_2019_4347_MOESM1_ESM.docx]

**Table S1 -** Linear Regression Model predicting the degree of change (with 95% CI) in HU of VAT and SAT density

|  | **Visceral adipose tissue density (HU)** | |  | **Subcutaneous adipose tissue density (HU)** | |
| --- | --- | --- | --- | --- | --- |
|  | **Crude  β-coefficient [95%CI]** | **Adjusted  β-coefficient [95%CI]*** |  | **Crude  β-coefficient [95%CI]** | **Adjusted  β-coefficient [95%CI]**** |
| **Prior exposure to TA and/or ddI** | -3.7 [-4.5; -2.8] | -1.4 [-2.2; -0.6] |  | -1.8 [-2.5; -1.0] | -1.8 [-2.5; -1.0] |
| **Age, per 10 years** | -2.1 [-2.4; -1.7] | -0.6 [-1.0; -0.2] |  | -0.9 [-1.2; -0.5] | -0.6 [-1.0; -0.3] |
| **Sex** | -1.2 [-2.6; 0.1] | -0.2 [-1.4; 1.0] |  | 0.9 [-0.2; 2.1] | -3.4 [-4.6; -2.3] |
| **VAT area, per 50cm^2^ increase** | -3.0 [-3.2; -2.7] | -2.3 [-2.7; -2.0] |  | - | - |
| **SAT area, per 50cm^2^ increase** | - | - |  | -1.6 [-1.8; -1.4] | -2.6 [-3.0; -2.3] |
| β coefficients represent the degree of change in HU of VAT and SAT density, respectively, for every 1-unit of change in the explanatory variables.  **Abbreviations**: visceral adipose tissue, VAT; subcutaneous adipose tissue, SAT; thymidine nucleoside analog reverse-transcriptase inhibitors, TA; didanosine, ddI.  Multivariable models were adjusted for: age, sex, origin, physical activity, BMI, smoking, VAT* (or SAT**) area, and prior exposure to TA and/or ddI | | | | | |
